# Minimal residual disease detection by multicolor flow cytometry in cryopreserved ovarian tissue from leukemia patients

**DOI:** 10.1186/s13048-021-00936-4

**Published:** 2022-01-18

**Authors:** Tristan Zver, Sophie Frontczak, Catherine Poirot, Aurélie Rives-Feraille, Brigitte Leroy-Martin, Isabelle Koscinski, Francine Arbez-Gindre, Francine Garnache-Ottou, Christophe Roux, Clotilde Amiot

**Affiliations:** 1 CHU de Besançon, Service de Biologie et Médecine de la Reproduction, Cryobiologie, CECOS Bourgogne Franche-Comté, 25000 Besançon, France; 2 Univ. Bourgogne Franche-Comté, INSERM, EFS BFC, UMR1098, RIGHT Interactions Hôte-Greffon-Tumeur/Ingénierie Cellulaire Et Génique, 25000 Besançon, France; 3 INSERM CIC-1431, CHU Besançon, 25000 Besançon, France; 4 Hôpital Saint-Louis, Service d’Hématologie, Unité AJA, 75010 Paris, France; 5 CHU de Rouen, Centre d’Assistance Médicale à la Procréation CECOS, 76000 Rouen, France; 6 CHU de Lille, Laboratoire de Biologie de la Reproduction, CECOS, Spermiologie, 59000 Lille, France; 7 CHRU de Nancy, Service de Biologie de la Reproduction, CECOS, 54035 Nancy, France; 8 CHU de Besançon, Service d’Anatomie Pathologique, 25000 Besançon, France

**Keywords:** Multicolor flow cytometry, Minimal residual disease, Ovarian tissue cryopreservation, Fertility preservation

## Abstract

**Background:**

Cryopreservation of ovarian tissue is a fertility-preservation option for women before gonadotoxic treatments. However, cryopreserved ovarian tissue transplantation must be performed with caution in women with malignancies that may metastasize to the ovaries. For this purpose, detecting minimal residual disease (MRD) in the ovarian cortex using sensitive methods is a crucial step. We developed an automated ovarian tissue dissociation method to obtain ovarian cell suspensions.

**Results:**

We assessed MRD by multicolor flow cytometry (MFC) in cryopreserved ovarian cortex of 15 leukemia patients: 6 with B-cell acute lymphoblastic leukemia (B-ALL), 2 with T-cell acute lymphoblastic leukemia (T-ALL) and 7 with acute myeloid leukemia (AML). Ovarian MRD was positive in 5 of the 15 leukemia patients (one T-ALL and 4 AML). No B-ALL patient was positive by MFC. Quantitative reverse-transcribed polymerase chain reaction was performed when a molecular marker was available, and confirmed the MFC results for 3 patients tested. Xenografts into immunodeficient mice were also performed with ovarian cortical tissue from 10 leukemia patients, with no evidence of leukemic cells after the 6-month grafting period.

**Conclusions:**

In conclusion, this is the first study using MFC to detect MRD in ovarian cortical tissue from acute leukemia patients. MFC has been accepted in clinical practice for its ease of use, the large number of parameters available simultaneously, and high throughput analysis. We demonstrate here that MFC is a reliable method to detect MRD in cryopreserved ovarian tissue, with a view to controlling the oncological risk before ovarian tissue transplantation in leukemia patients.

**Supplementary Information:**

The online version contains supplementary material available at 10.1186/s13048-021-00936-4.

## Background

Overall cancer incidence for patients aged 0–19 years has been increasing steadily since the 1970s, but death rates are continuously decreasing, with a 5-year survival rate for all cancers higher than 83% [1]. This results in an increasing number of childhood cancer survivors [2, 3] for whom reproductive health is a major concern [4]. Cancer treatments such as chemotherapy and radiotherapy, are known to be gonadotoxic (especially alkylating agents), and lead to premature ovarian failure and infertility in some circumstances [5–7].

To date, several options have been used to preserve and restore fertility in female patients [8]. Many international guidelines for fertility preservation and restoration have been published [9–14]. Embryo and oocyte cryopreservation are well-established techniques. Ovarian tissue cryopreservation (OTC) has only recently been classed as an established procedure by the American Society for Reproductive Medicine [14], according to reports in the literature of successful reuse of ovarian cortical tissue with subsequent live births [15–20]. For the European Society for Medical Oncology and European Society of Human Reproduction and Embryology, OTC is an alternative and recommended procedure when embryo/oocyte cryopreservation is not feasible [21, 22], whereas the American Society for Clinical Oncology stilled considered it experimental as of 2018 [10]. Ovarian cortex autograft is currently the only way to re-use frozen/thawed ovarian cortical strips.

Leukemia is the most common form of cancer in children, adolescents and young adults [23]. The majority of these patients are prepubertal girls, and they cannot delay the start of chemotherapy and/or radiotherapy. OTC is therefore the only option to preserve fertility in these patients [24–26]. However, in case of cancers with a high risk of ovarian metastasis, such as acute leukemia, an important concern is the risk of relapse via the graft. An autopsy study performed in Japan reported the presence of ovarian metastasis in 8.4% of leukemia patients under the age of 40 [27].

In hematology laboratories, two methods are currently used for MRD monitoring in blood or bone marrow, namely polymerase chain reaction (PCR) amplification of specific transcripts or Ig gene rearrangements, and detection of leukemia-associated immunophenotype by multicolor flow cytometry (MFC) [28–30]. Some studies have investigated the detection of minimal residual disease (MRD) in cryopreserved ovarian tissue using molecular techniques [31–40], xenograft of cryopreserved ovarian tissue in immunodeficient mice [34, 35, 39, 41] and next-generation sequencing [41] to highlight the presence of malignant cells. To detect MRD in ovarian tissue by MFC, it is necessary to identify markers that enable the differentiation of leukemic cells from viable ovarian cells by the use of leukemia-associated immunophenotypes (LAIP).

Our team has developed and validated a technique to detect leukemic cells in the ovarian cortex of patients with acute lymphoblastic leukemia (ALL) [42] or acute myeloid leukemia (AML) [43] via MFC, using a standardized protocol for ovarian cortex dissociation [44].

The present study investigated the presence of leukemic cells in cryopreserved ovarian cortex from patients with B-cell acute lymphoblastic leukemia (B-ALL), T-cell acute lymphoblastic leukemia (T-ALL) and AML. Detection of MRD was carried out by MFC, quantitative reverse-transcribed polymerase chain reaction (RT-qPCR) when a molecular marker was available and xenograft in immunodeficient mice.

## Methods

### Patients

Frozen/thawed ovarian cortical tissue was obtained from 15 leukemia patients: 6 with B-ALL, 2 with T-ALL and 7 with AML (Table 1). For 7 patients, cryopreservation of ovarian cortical tissue was performed in Besançon university hospital and for 8 patients, in 4 other university hospitals in France (Lille, Nancy, Paris and Rouen). Ovarian cortical tissue was transported from these centers in vapor nitrogen and stored in the cryobank of the Assisted Reproductive Technology Center of Besançon university hospital before MRD testing.

**Table 1:** Patient and pathology characteristics

Patient no	Age at OTC	Type of leukemia	Treatment received before OTC				LAIP identified at diagnosis	Molecular marker at diagnosis
			IV, IM or per os	IT	CED	DIE		
1	29	B-ALL	1, 2, 11, 15, 18	1, 11, 14	0	0	CD10 (100%), CD19 (100%), CD20 (87%0, CD22 (100%), CD34 (78%), CD38 (100%), CD58 (100%), CD200 (62%), CD304 (78%), cMPO (81%), oCD22 (99%), cTDT (73%), cCD79a (99%)	BCR-ABL1
2	31	B-ALL	1, 2, 3, 4, 5, 6, 11, 12	1, 11, 14	2 500	175	CD19 (100%), CD22 (96%), cyCD22 (94), CD34 (100%), CD38 (99%), CD44 (98%), CD58 (98%), CD123 (99%), cyTDT (90%), cyCD79a (94%)	Ig/TCR
3	14	B-ALL	1, 2, 3, 5, 6, 7, 8, 9, 10, 11, 12, 14, 15	1, 11, 14	1 464	191	CD19 (91%), CD34 (87%), negative for CD10 and myeloid markers	Ig/TCR
4	12	B-ALL	1, 2, 3, 5, 6, 11	1, 11, 19	0	24	CD45 (88%), HLA DRII (88%), CD10 (78%), CD19 (78%), CD22 (82%), CD33 (35%)	Ig/TCR
5	5	B-ALL	1, 2, 3, 4, 5, 6, 7, 9, 19, 11, 12, 13, 14, 15	1, 11, 14, 19	2 500	314	CD19 (86%), CD10 (98%), CD22 (90%) CD38 (99%)	Unkown
6	5	B-ALL	1, 2, 3, 6, 7, 8, 9, 10, 11, 12, 14, 15	1, 11, 19	610	87	CD45 (12%), CD10 (87%), CD19 (79%), CD22 (86%), CD34 (70%) HLA-DRII (81%)	Ig/TCR
7	14	T-ALL	1, 2, 3, 4, 6, 11, 12, 15, 20	1, 11, 14	2 000	100	CD2 (89%), cyCD3 (94%), CD5 (93%), CD7 (98%), CD10 (49%), CD33 (81%), CD34 (89%), CD45RA (99%), CD123 (56%)	None
8	22	T-ALL	1, 2, 3, 4, 6, 16	1, 11, 14	1 000	133	CD2 (76%), CD7 (94%), CD13 (98%), CD10 (80%), cyCD3 (83%), cyCD79 (80%)	None
9	33	AML	1, 3, 17	1, 11, 14	0	300	CD13 (100%), cyCD13 (100%), CD15 (55%), CD33 (100%), CD34 (100%), CD38 (100%), CD117 (100%), cMPO (100%)	CBFB-MYH11
10	15	AML	1	1, 11, 19	0	373	CD34 (96%), CD33 (98%), CD13 (54,5%), CD117 (47%), CD38 (40,5%)	WT1
11	22	AML	1, 3, 17	1, 11,14	0	300	CD7 (80%), CD11b (83%), CD13 (96%), cCD13 (66%), CD19 (51%), CD33 (96%), CD34 (100%), CD38 (100%), CD71 (93%), CD117 (100%), cyMPO (100%), HLA DR (92%)	WT1
12	15	AML	1, 13	1, 11, 19	0	240	CD13 (90%), CD33 (99%), CD117 (99%), CD65 (86%), CD7 (99%), HLA DR2 (97%), CD34 (98%), CD38 (98%)	CEBPA mutations
13	14	AML	1, 13, 16	1, 11, 19	0	48	CD13 (100%), CD33 (100%), CD65 (100%), CD117 (100%), et CD11c (100%)	Unknown
14	26	AML	None	None	0	0	CD13 (100%), CD33 (100%), CD117 (100%), CD34 (100%), CD38 (100%), CD123 (89%), cyMPO (100%), cyCD13 (100%)	None
15	27	AML	1, 4, 14, 15, 16, 17, 21	None	0	225	CD34 (100%), CD33 (50%), CD38 (100%), HLA DR (100%), CD99 (50)	BCR-ABL1

*OTC* ovarian tissue cryopreservation, *MRD* minimal residual disease, *LAIP* leukemia-associated immunophenotype, *IV* intravenous, *IM* intramuscular, *IT* intrathecal, *CED* cyclophosphamide equivalent dose, *DIE* doxorubicin isotoxic equivalent, *B-ALL* B-cell acute lymphoblastic leukemia, *T-ALL* T-cell acute lymphoblastic leukemia, *AML* acute myeloid leukemia, *Ig* immunoglobulin rearrangement genes, *TCR* T-cell receptor rearrangement genes

1 indicates cytarabine, 2 vincristine, 3 daunorubicin, 4 cyclophosphamide, 5 etoposide, 6 asparaginase, 7 doxorubicin, 8 ifosfamide, 9 thioguanine, 10 vindesine, 11 methotrexate, 12 mercaptopurine, 13 mitoxantrone, 14 prednisolone, 15 dexamethasone, 16 amsacrine, 17 idarubicin, 18 imatinib, 19 hydrocortisone, 20 vinblastine, 21 gemtuzumab

See Additional file 1 for CED and DIE calculation

Slow-freezing cryopreservation of ovarian cortical tissue was performed between 2004 and 2018. For 3 patients, the medulla was also cryopreserved after dissection from the ovarian cortex. Patients were aged between 5 and 31 years old when ovarian tissue cryopreservation was performed. The mean age was 18.9 years at the time of cryopreservation.

All leukemia patients received chemotherapy before OTC except one AML patient (patient 14). Chemotherapy drugs and cumulative doses of alkylating agents (cyclophosphamide equivalent doses) [45] and anthracyclines (doxorubicin isotoxic doses) [46] are listed in Table 1. For each patient, the LAIP found in the blood or bone marrow at diagnosis was used for MRD detection in frozen/thawed ovarian tissue. Any molecular markers identified at diagnosis are listed in Table 1.

### Ovarian tissue cryopreservation and thawing

Cortical biopsies were cryopreserved in cryovials containing freezing solution consisting of 1.5 M dimethyl sulfoxide (DMSO; Sigma) and 0.1 M sucrose (Sigma) in Leibovitz’s L-15 medium (Eurobio) supplemented with 10% heat-inactivated patient serum, according to a protocol using slow cooling with manual seeding as previously published [47]. After freezing, the vials were stored in liquid nitrogen. All ovarian cortical biopsies were thawed according to a previously described technique [48]. The vials were warmed at room temperature for 30 s, then immersed in a 37 °C heat chamber (5 min), and the ovarian tissue pieces were washed in decreased solutions of DMSO 1.5 M (5 min), 1 M (5 min) and 0.5 M (10 min) and in a solution of Leibovitz’s L-15 medium supplemented with 20% heat-inactivated AB serum from blood donors (10 min).

### Isolation procedure for ovarian cells

Ovarian cortex was cut into small pieces of 1–2 mm^3^. Depending on the timing of the MRD test, a so-called “laboratory” protocol (as previously described) [44] or a commercial protocol was used for ovarian cell isolation. Briefly, the laboratory protocol is based on mechanical and enzymatic dissociation using a cell dissociator (GentleMACS; Miltenyi Biotec) and collagenase Ia (1.6 U/ml; Sigma) with DNase I (Roche) in 5 ml of RPMI (ThermoFisher Scientific) in combination with C Tubes (Miltenyi Biotec). For the commercial protocol, a Tumor Dissociation Kit was used according to the manufacturer’s instructions (Miltenyi Biotec); it was previously validated in our laboratory for ovarian tissue dissociation [44]. After ovarian tissue dissociation, we performed cell suspension filtration with a 70 µm cell strainer (Dutscher) and washed the suspension with 5 mL of RPMI. Next, the suspension was centrifuged at 300 g for 7 min and the pellet was resuspended in the appropriate volume.

### Multicolor flow cytometry detection of MRD

Eight-color MFC was performed using a BD CANTO II flow cytometer (BD Biosciences) and data were acquired and analyzed using Diva and Flowjo software (BD Biosciences), respectively. The compensation matrix was set up using calibration beads (compbeads®, BD Biosciences) according to the manufacturer’s instructions.

The same combinations of eight monoclonal antibodies (mAbs) applied to leukemic cells at diagnosis (when available) were used for ovarian cell suspension from leukemia patients for MRD assessment. The panel used was composed of 4 fixed dye/mAbs and 4 variable dye/mAbs determined according to the leukemia patient’s LAIP. Accordingly, it was used as follows: 7-AAD (Beckman Coulter) and SYTO13 (ThermoFisher Scientific) are used to identify nucleated viable cells (7-AAD^−^/SYTO13^+^ phenotype), CD45-V500 (HI30, BD Biosciences) or CD45-BV510 (Brillant Violet 510™, HI30, BD Biosciences) to characterize leucocytes (CD45^+^) and CD3-V450 (UCHT1; BD Biosciences) or CD3-BV421 (Brillant Violet 421™, UCHT1, BD Biosciences) or APCH7 (Allophycocyanin H7, SK7, BD Biosciences) to isolate residual T lymphocytes (CD45^+^/CD3^+^ phenotype). For patient 15, we used FVS 780 (BD Horizon™ Fixable Viability Stain 780, BD Biosciences) and CD45-PerCP-Cyanine5.5 (HI30, BD Biosciences) in place of 7-AAD and CD45-BV510, respectively. The 4 variable mAbs used were determined based on the patient’s LAIP at diagnosis, and low or non-expression of their antigen target by normal ovarian cells or mature lymphocytes. This enabled us to select the best panel for MRD assessment in ovarian cortical tissue (See Additional file 1). For patients 7 and 8, a preliminary step of fixation and permeabilization (IntraStain, Dako) was required for cytoplasmic detection of CD3 (cyCD3). Cells were labelled as previously described. Briefly, antibodies were incubated with cells for 20 min at 4 °C followed by centrifugation at 300 g for 7 min to eliminate excess antibodies. Pelleted cells were then resuspended in 100 µl of PBS for acquisition.

Viable cells (7-AAD^−^/SYTO13^+^ or FVS 780^−^/SYTO13^+^) were used to calculate the MRD level. Among the viable cells, CD45^+^ leukocytes and CD3^+^ T lymphocytes were identified. The threshold of 10^–4^ (1 cell in 10,000) is currently used in immunohematology laboratories to define MRD positivity in blood or bone marrow, and was used by default to define MRD positivity in ovarian cortical tissue for the purposes of this study.

### Xenotransplantation

Female (CD-1® Nude) immunodeficient mice were obtained from Charles River Laboratories (France). They were kept at 4 per individually ventilated cage, with free access to food and water. At 7 weeks of age, the mice were anesthetized with isofluran (Baxter). One strip of frozen/thawed ovarian cortex from each leukemia patient was divided into equal small pieces (around 0.25 cm^2^) and transplanted subcutaneously into two immunodeficient mice on the right or left side of the vertebral column. At 24 weeks post-transplantation, mice were sacrificed by cervical dislocation to harvest the ovarian grafts (when visible) and specific organs (femur, lymph nodes, spleen, blood) which were then manually dissociated for MRD analysis by MFC. We used a rat anti-mouse CD45-V450 (30-F11, BD Biosciences) to distinguish murine cells from human cells by MFC.

## Results

### Validation of the gating strategy by MFC

**Fig. 1:**
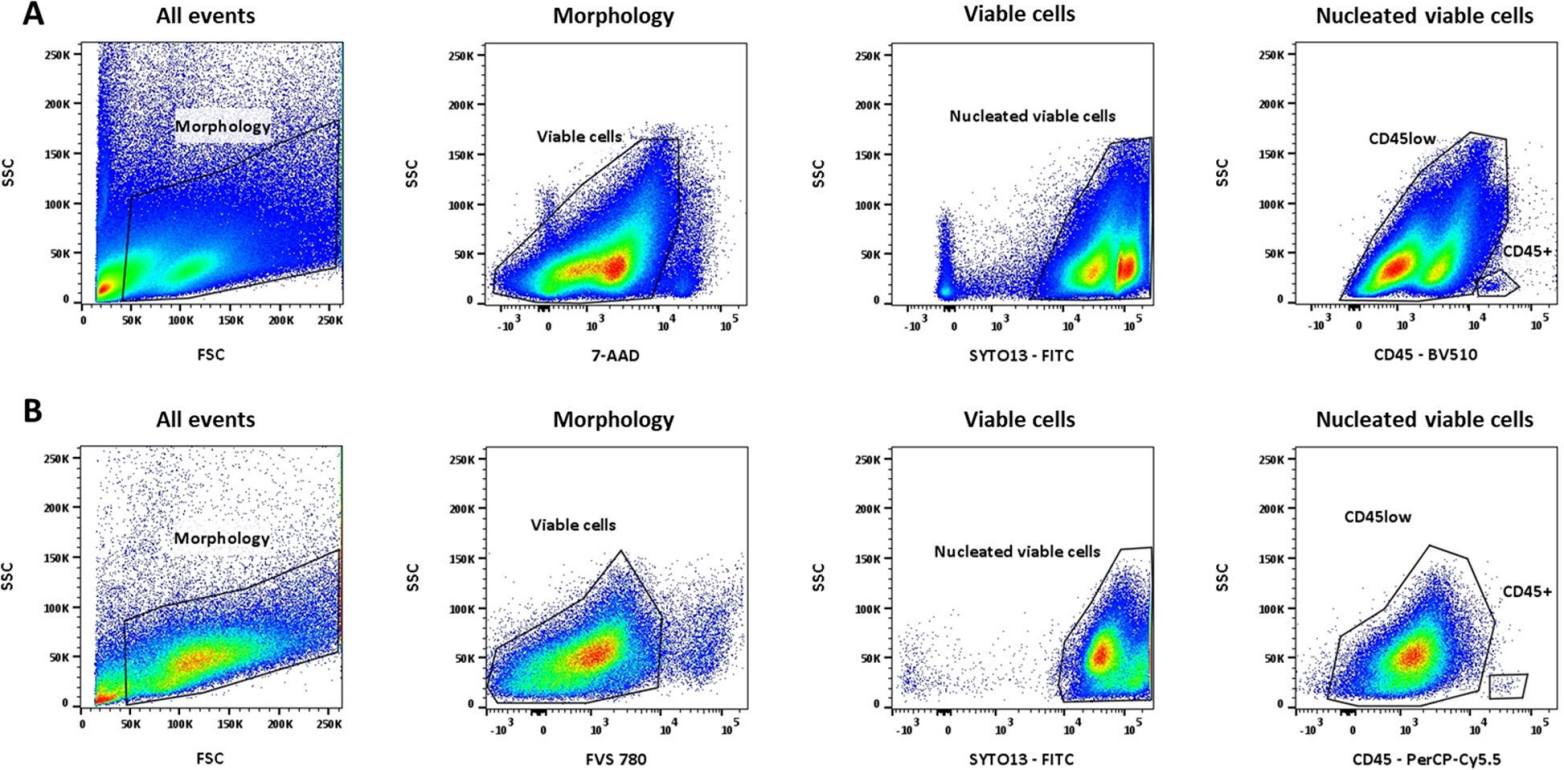
MFC gating strategy applied to detect MRD in ovarian samples. The observed populations are indicated at the top of the dot plots. The first gate is used for debris exclusion using SSC and FCS light scatter (Morphology). The 7-AAD^−^ or FVS780^−^ combined with SYTO13^+^ set the nucleated viable cells. CD45 enabled us to separate CD45^+^ leucocytes from other viable cells for MRD analysis. **A** Gating strategy with 7-AAD. **B** Gating strategy with FVS780. Data acquired with Diva software and analysed with Flowjo software

This study confirmed that ovarian cells can be identified based on the elimination of debris by using side (SSC, for granularity) and forward (FSC, for size) light scatter characteristics, and 7-AAD^−^/SYTO13^+^ (Fig. 1A) or FVS 780^−^/SYTO13^+^ (Fig. 1B) for the viable ovarian cells. CD45^low^ cells correspond to viable ovarian cells or leukemic cells, while CD45 positive cells can be identified as leucocytes (Fig. 1A and B).

### MRD detection by MFC

Of the 15 patients, all could be analyzed by MFC to detect MRD in ovarian cortical tissue. Eight patients displayed molecular markers suitable for MRD detection, while 7 patients had no markers, or had unknown or unusable markers (for example, WT1 for patients 10 and 11).

**Fig. 2:**
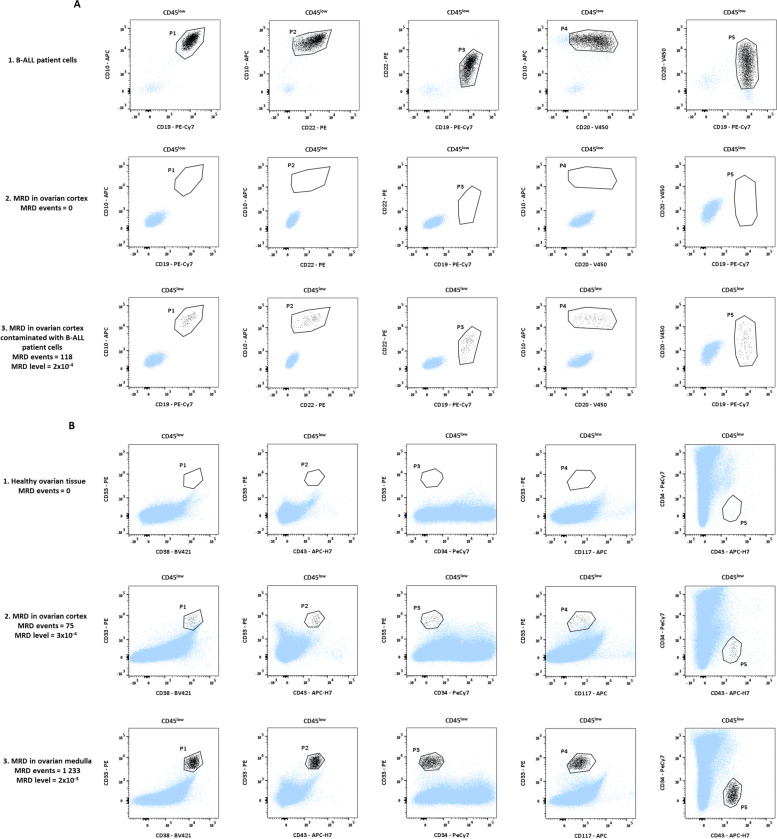
MRD detection by MFC in ovarian tissue from leukemia patients. The observed populations are indicated at the top of the dot plots (CD45^low^, see Fig. 1). **A** B-ALL patient (patient 1) with negative MRD in the ovarian cortical tissue. (1) B-ALL cells at diagnosis express the following immunophenotype: CD19^+^/CD10^+^/CD22^+^/CD20^+low^. (2) Ovarian cells from patient 1: in 449 438 events acquired, we identified no event presenting the same phenotype as the B-ALL cells at diagnosis (P1 ∩ P2 ∩ P3 ∩ P4 ∩ P5). (3) Ovarian cells from patient 1 artificially contaminated with B-ALL cells: in 540 035 events acquired (CD45^low^ events), we identify 118 events presenting the same phenotype as the B-ALL cells (P1 ∩ P2 ∩ P3 ∩ P4 ∩ P5): the artificial MRD level is quantified at 2.2 × 10^–4^. **B** AML patient (patient 14) with positive MRD in the ovarian tissue. (1) Healthy ovarian tissue (control): there is no event presenting an AML immunophenotype (CD33^+^/CD38^+^/CD34^low^/CD117^+low^). (2) Ovarian cortical cells from patient 14: in 267 702 events acquired, we identified 75 events presenting the same phenotype as the AML cells at diagnosis (P1 ∩ P2 ∩ P3 ∩ P4 ∩ P5). The MRD level is quantified at 2.8 × 10^–4^. (2) Ovarian medulla cells from patient 14: in 738 895 events acquired, we identified 1 233 events presenting the same phenotype as the AML cells at diagnosis (P1 ∩ P2 ∩ P3 ∩ P4 ∩ P5). The MRD level is quantified at 1.7 × 10^–3^

Among the B-ALL patients (*n* = 6, patients 1 to 6), none was found to positive by MFC for MRD in their ovarian cortical tissue (Table 2). Two patients were also negative for ovarian MRD by RT-qPCR, confirming the results obtained by MFC. Three other B-ALL patients had Ig/TCR rearrangements at diagnosis, but we did not have the technologies in our laboratory to test ovarian cortical tissue from these patients by this method at diagnosis or prior to OTC. The sixth patient had no molecular marker to detect MRD. For patient 1, no MRD positive event was detected among 449 438 total viable nucleated events (Fig. 2A) with a maximum sensitivity for the experiment of 4.4 × 10^–5^ (Table 2). When the ovarian cortical tissue from this patient was artificially contaminated with B-ALL patient cells at diagnosis, we were able to detect these cells by MFC (Fig. 2A), confirming the ability of MFC to identify leukemic cells in ovarian tissue.

**Table 2:** MRD results of ovarian cortical tissue obtained by MFC and RT-qPCR

Patient no	Type of leukemia	Ovarian cortical tissue					Ovarian cortical tissue xenografts			
		MFC				RT-qPCR	MFC			
		Viable events	Positive events	MRD level		MRD level	Bone marrow	Blood	Spleen	Lymph nodes
1	B-ALL	449 438	0	Negative < 5 × 10^–5^	Negative Undetectable		Negative < 4 × 10^–5^	Negative < 6 × 10^–5^	Negative < 4 × 10^–5^	Negative < 2 × 10^–4^
2	B-ALL	295 103	3	Negative < 7 × 10^–5^	Negative < 1.10^–4^		Negative < 4 × 10^–5^	Negative < 8 × 10^–5^	Negative < 4 × 10^–5^	Negative < 3 × 10^–4^
3	B-ALL	333 314	1	Negative < 6 × 10^–5^	NP		Negative < 3 × 10^–5^	Negative < 4 × 10^–5^	Negative < 3 × 10^–5^	Negative < 5 × 10^–5^
4	B-ALL	336 425	0	Negative < 6 × 10^–5^	NP		Negative < 3 × 10^–5^	Negative < 2 × 10^–4^	Negative < 3 × 10^–5^	Negative < 8 × 10^–5^
5	B-ALL	233 918	0	Negative < 9 × 10^–5^	NA		Negative < 5 × 10^–5^	Negative < 2 × 10^–5^	Negative < 5 × 10^–5^	Negative 7 × 10^–5^
6	B-ALL	233 868	0	Negative < 9 × 10^–5^	NP		Negative < 2 × 10^–5^	Negative < 5 × 10^–5^	Negative < 5 × 10^–5^	Negative < 8 × 10^–5^
7	T-ALL	272 007	1	Negative < 8 × 10^–5^	NA		NP	NP	NP	NP
8	T-ALL	1,360,814	335	Positive 3 × 10^–4^	NA		Negative < 6 × 10^–5^	Negative < 1 × 10^–4^	Negative < 3 × 10^–5^	Negative < 1 × 10^–4^
9	AML	254 777	7	Negative < 8 × 10^–5^	Negative Undetectable		NP	NP	NP	NP
10	AML	331 675	1	Negative < 6 × 10^–5^	NP		Negative < 3 × 10^–5^	Negative < 9 × 10^–5^	Negative < 4 × 10^–5^	Negative < 7 × 10^–5^
11	AML	385 545	69	Positive 2 × 10^–4^	NP		Negative < 3 × 10^–5^	Negative < 8 × 10^5^	Negative < 3 × 10^–5^	Negative < 1 × 10^–4^
12	AML	133 216	34	Positive 3 × 10^–4^	NP		Negative < 2 × 10^–5^	Negative < 7 × 10^–5^	Negative < 4 × 10^–5^	Negative < 6 × 10^–5^
13	AML	231 193	85	Positive 4 × 10^–4^	NA		NP	NP	NP	NP
14	AML	267 702	75	Positive 3 × 10^–4^	NA		NP	NP	NP	NP
15	AML	55 391	0	Negative < 4 × 10^–5^	NP		NP	NP	NP	NP

Data present the number of leukemia patients and type of leukemia. Results of MRD detection in ovarian cortical tissue and after grafting experiments by MFC and RT-qPCR when possible are also presented. MRD values were obtained by dividing the number of cells in the LAIP gate (positive events) by the total number of viable events analysed. To assess the maximum of sensitivity corresponding to the limit of detection that can be achieved for each MRD experiment, we divided the minimal number of cells to define a significant abnormal cell population (20 LAIP^+^ cells) by the number of viable events analysed in this experiment

*B-ALL* B-cell acute lymphoblastic leukemia, *T-ALL* T-cell acute lymphoblastic leukemia, *AML* acute myeloid leukemia, *MFC* multicolor flow cytometry, *RT-qPCR* reverse transcription-quantitative polymerase chain reaction, *NA* not available, *NP* not performed

Among the T-ALL patients (*n* = 2, patients 7 and 8), ovarian cortical tissue from one patient was detected positive by MFC (patient 8). Indeed, we identified 335 LAIP positive events among 1.36 × 10^6^ viable events. MRD for patient 8 is thus positive at a level of 3 × 10^–4^ (Table 2).

Among the AML patients (*n* = 7, patients 9 to 15), 4 patients showed positive MRD by MFC in their ovarian cortical tissue (Patient 11, 12, 13 and 14) (Table 2). For patient 9, the MFC result was confirmed negative by RT-qPCR. RT-qPCR was not performed for the other AML patients due to unsuitable markers in the ovarian cortex (Patients 10 and 11), lack of ovarian cortical tissue for testing (Patients 12 and 15) or no molecular marker available or known for MRD detection (Patients 13 and 14). Medulla was tested by MFC for patients 11, 14 and 15: the results confirmed those obtained in ovarian cortical tissue, i.e. positive MRD for patients 11 and 14, and negative MRD for patient 15. MRD results obtained in the medulla and in the cortical tissue of patient 14 are presented in Fig. 2B.

Among the 5 patients who were positive for MRD in their ovarian tissue by MFC, 4 had already received chemotherapy before OTC: 3 had undergone one regimen of induction and consolidation, and 1 patient only one regimen of induction (Table 1). These results confirm that firstline chemotherapy is not completely toxic to malignant cells, and leukemia patients in complete remission may harbor leukemic cells in their ovarian cortical tissue.

### Xenotransplantation

Twenty nude mice (two per patient) were grafted with ovarian cortical tissue from B-ALL patients (*n* = 6, patients 1 to 6), one T-ALL patient (*n* = 1, patient 8) or AML patients (*n* = 3, patients 10 to 12). Ovarian cortical tissue from patients 7, 9, 12, 13 and 14 was not tested by xenotransplantation as there was insufficient tissue available.

One mouse died during the 24 weeks of the xenografting period because of weight loss (patient 3). None of the other mice showed any macroscopic signs of malignancy (e.g., weight loss, enlarged organs). After 24 weeks of grafting, spleen, lymph nodes, blood and bone marrow were recovered from all mice for MFC analysis.

Human grafted ovarian cortical tissue was not found on autopsy of both mice for 4 patients (patients 1, 2, 5 and 12), and was not found on autopsy of one mouse for 4 patients (patients 3, 4, 8 and 10). No proliferation or tumor growth was observed on xenografted ovarian cortical tissue. Pieces of ovarian cortical tissue were reduced in size during the xenografting experiment, making MRD detection by MFC impossible.

For all patients, serial sections of grafts and organs were observed and did not show any signs of malignant cells (data not shown). The search for MRD by MFC revealed no leukemia cells in the bone marrow, blood, lymph nodes or spleen of mice (Table 2). RT-qPCR was performed for four patients (BCR-ABL1 for patient 1 and WT1 for patients 10, 11 and 12) on different organs when there was sufficient material. All results were negative (data not shown), confirming the results obtained by MFC.

## Discussion

To the best of our knowledge, this is the first study in a cohort of leukemia patients where MFC is used to detect MRD in ovarian cortical tissue. This technology has previously been validated by our team, demonstrating its effectiveness for the detection of MRD in ovarian cortical tissue [42, 43, 49].

Ovarian cortical tissue cryopreservation is currently the only available method to preserve fertility for prepubertal children or women who cannot delay chemo- and/or radiotherapy [8, 24]. Indeed, ovarian cortex transplantation is the only established technique for re-use of ovarian cortex, with a high success rate [15, 17, 18, 20, 50–52]. Our team has set up a study in France called DATOR (Development of Ovarian Tissue Autograft in Order to Restore Ovarian Function) (NCT02846064) with the aim of assessing the safety and efficacy of ovarian cortex transplantation [19]. However, in leukemic patients, this technique incurs a risk, with the possibility of cancer reseeding. It is therefore important to develop techniques for MRD detection in ovarian cortical tissue.

Among 15 leukemia patients included in this study, ovarian cortical tissue was positive for MRD by MFC in 5 of them (33%). Results obtained by MFC were confirmed where possible by RT-qPCR (in 3 patients). Molecular markers were available for other patients, but analysis was not performed due to lack of ovarian cortex. However, molecular analysis could be performed just before autotransplantation of ovarian cortex to confirm MFC results, as was done in patient 2, for example. The findings presented in this study are congruent with previous reports from other teams [34, 35, 38, 40].

Xenograft studies failed to amplify leukemic cells identified by MFC. These results corroborate those reported by a Danish team [35], where no MRD amplification was observed, but contrast with those from a Belgian team [34], who observed clinical disease. Another study, published by Diaz-Garcia in 2019, also showed MRD amplification by a xenograft model [53]. However, this model for MRD detection is time consuming, and depends on multiple factors, such as the mouse model [54] (SCID, Nude, NSG for example), or the graft site [55], which can explain the difficulty of reproducing results. The major conclusion of all these studies is that they confirm the potential for leukemic cell contamination in ovarian cortical tissue [34–36, 53], but that positive MFC results do not necessarily translate into disease recurrence.

Currently, we do not know the level of MRD that can induce relapse after ovarian cortical tissue transplantation. Results differ between studies. Injection of 200 leukemic cells into nude mice was shown to induce leukemia in one study [56], whereas 1000 cells were unable to induce relapse in another [57]. In one recent study, malignant cells were found in mice injected with 1000 cells, and clinical disease was only caused by injection of 5 × 10^6^ leukemic cells [53], in line with the findings of a previous study [57]. Host species and grafting site, as well as the heterogeneity of leukemic contamination in ovarian cortical tissue, may explain these discrepancies between studies [53, 58]. It is also important to bear in mind that a patient who receives ovarian cortical tissue transplantation has an immune system to fight leukemic cells, contrary to immunodeficient mice. The minimal dose of transplanted leukemic cells that may lead to leukemia in mouse or human recipients remains unknown, and therefore, the established flow cytometric platform cannot currently be used as a selection criterion for suitability of autografting. Further research is needed to identify the threshold of leukemic cells that could induce cancer relapse in patients. Future studies could provide important information on the relationship between the relative and absolute number of leukemic cells in an ovarian autograft and the clinical outcome after its transplantation. This may ultimately determine whether it is really necessary to assess leukemia MRD in ovarian tissue grafts.

Despite the risk related to ovarian cortical tissue transplantation in case of leukemia, six live births have been reported in the literature after ovarian cortex transplantation in leukemia patients [18, 41, 59]. In each case, the search for MRD in ovarian cortical tissue was done with reliable techniques like histology, molecular techniques, next generation sequencing or xenograft into immunodeficient mice. However, these tests are time-consuming, expensive and hard to achieve for most laboratories (need for experience and facilities for animal experimentation).

MFC can also potentially be adapted to all leukemia patients with LAIP, contrary to PCR, which is potentially applicable in 28–89% of patients [41]. MFC has been used with success by other teams on ovarian cortical tissue [60, 61]. Many hospitals have a hematology laboratory, where the leukemia diagnosis is made. It is also easy to obtain the diagnostic information to constitute LAIP for MRD investigation in ovarian cortical tissue. When using MFC, the ideal method is to use leukemic blasts frozen at diagnosis to test the antibody panel on these cells, and reference ovarian tissue with no leukemic cells. The MFC technique can be implemented rapidly, contrary to xenografting into immunodeficient mice, for example. Nevertheless, it is important to perform reliable techniques to assess and confirm MRD results obtained by other methods. MRD evaluation in the residual medulla, when available, is recommended by ESHRE [22]. In our study, MRD results for the cortex and medulla were concordant in 3 patients, and in line with a recent study where 20/24 MRD results were concordant [40].

Whether chemotherapy is received before OCT or not does not seem to have any impact on ovarian cortical tissue MRD results. Indeed, in our study, positive MRD was observed in patients who received treatment, and in one patient without chemotherapy. The treatment received by the patients before OTC was at low risk in terms of gonadotoxicity, with a Cyclophosphamide Equivalent Dose < 4 000 mg/m^2^ (0—2 500 mg/m^2^) and a Doxorubicin Isotoxic Equivalent > 250 mg/m^2^ for 4/15 patients (cardiac toxicity) [62]. Some studies have reported that exposure to chemotherapy before OTC does not alter the future result of ovarian cortical tissue transplantation [50, 63, 64]. However, prior chemotherapy may decrease MRD in ovarian cortical tissue in leukemia patients [36]. As previously suggested by other groups, we recommend performing OTC after the first round of chemotherapy, or before hematopoietic stem cell transplantation, to reduce the risk of leukemic cells in ovarian cortical tissue.

In conclusion, cryopreserved ovarian cortex was positive for MRD by MFC in 5 out of 15 leukemia patients (4 AML and 1 T-ALL), even though RT-qPCR and/or xenograft MRD was negative for these patients, when performed. This study demonstrates that MFC is a reliable and easy-to-use technique to detect MRD in ovarian cortical tissue. This adds to the wide variety of techniques available to test ovarian MRD prior to transplantation in leukemia patients. This represents an important step to controlling oncological risk of ovarian cortex transplantation in leukemia patients. Whether MRD detection in ovarian cortical tissue has any clinical utility, and how the data should be incorporated in a clinical protocol will require follow-up studies in leukemia patients who have been transplanted with ovarian tissues having accurately documented MRD levels.

## Supplementary Information

Additional file 1: CED and DIE calculation, marker selection for MRD detection by MFC in ovarian tissue

## Data Availability

All data generated and analyzed during this study are included in the published article.
