# Supplementary material for: Minimal residual disease detection by multicolor flow cytometry in cryopreserved ovarian tissue from leukemia patients
Source: J Ovarian Res. 2022 Jan 18;15:9. doi: 10.1186/s13048-021-00936-4 (PMC8767661; doi:10.1186/s13048-021-00936-4)
Supplement: Supplementary file 1 — Additional file 1: CED and DIE calculation, marker selection for MRD detection by MFC in ovarian tissue [file 13048_2021_936_MOESM1_ESM.pdf]

Additionnal file 1:

CED and DIE calculation

Cyclophosphamide Equivalent Dose (CED) was calculated using the following equation:

CED (mg/m^2) = 1.0 (cumulative cyclophosphamide dose (mg/m^2)) + 0.244 (cumulative ifosfamide dose (mg/m^2)) + 0.857 (cumulative procarbazine dose (mg/m^2)) + 14.286 (cumulative chlorambucil dose (mg/m^2)) + 15.0 (cumulative BCNU dose (mg/m^2)) + 16.0 (cumulative CCNU dose (mg/m^2)) + 40 (cumulative melphalan dose (mg/m^2)) + 50 (cumulative Thio-TEPA dose (mg/m^2)) + 100 (cumulative nitrogen mustard dose (mg/m^2)) + 8.823 (cumulative busulfan dose (mg/m^2)) according to Green and colleagues[1].

Doxorubicin Isotoxic Equivalent (DIE) was calculated using the following formula:

DIE (mg/m^2) = = 1.0 (cumulative doxorubicin dose (mg/m^2)) + 0.833 (cumulative daunorubicin dose (mg/m^2)) + 5.0 (cumulative idarubicin dose (mg/m^2)) + 0.67 (cumulative epirubicin dose (mg/m^2)) + 4.0 (cumulative mitoxantrone dose (mg/m^2)) according to Shankar and colleagues[2].

References:

1. Green D. M., Liu W., Kutteh W. H., et al. Cumulative alkylating agent exposure and semen parameters in adult survivors of childhood cancer: a report from the St Jude Lifetime Cohort Study. *Lancet Oncol*. 2014;15(11):1215-1223.
2. Shankar S. M., Marina N., Hudson M. M., et al. Monitoring for cardiovascular disease in survivors of childhood cancer: report from the Cardiovascular Disease Task Force of the Children's Oncology Group. *Pediatrics*. 2008;121(2):e387-396.

Table : Markers selection for MRD detection by MFC in ovarian tissue.

Patient no.	LAIP identify in the blood or bone marrow at diagnosis	Markers selection for MRD detection in cortical tissue					
		V450/BV421	BV510	PE	PE-Cy7	APC	APC-H7
1	CD10 (100%), CD19 (100%), CD20 (87%), CD22 (100%), CD34 (78%), CD38 (100%), CD58 (100%),CD200 (62%), CD304 (78%), cMPO (81%), cCD22 (99%), cTDT (73%), cCD79a (99%)	CD20<br>L27 (BD)	/	CD22<br>S-HCL-1 (BD)	CD19<br>J3.119 (BC)	CD10<br>HI10a (BD)	/
2	CD19 (100%), CD22 (96%), cyCD22 (94%), CD34 (100%), CD38 (99%), CD44 (98%), CD58 (99%), CD123 (99%), cyTDT (90%), cyCD79a (94%)	CD34<br>581 (BD)	/	CD24<br>ML5 (BD)	CD19<br>J3.119 (BC)	CD123<br>AC145 (MB)	/
3	CD19 (91%), CD34 (87%), negative for CD10 and myeloid markers	/	/	CD24<br>ML5 (BD)	CD19<br>J3.119 (BC)	CD34<br>581 (BD)	/
4	CD45 (88%), HLA DRII (88%), CD10 (78%), CD19 (78%), CD22 (82%), CD33 (35%)	/	/	CD22<br>S-HCL-1 (BD)	CD19<br>J3.119 (BC)	CD10<br>HI10a (BD)	HLA-DR<br>G46-6 (BD)
5	CD19 (86%), CD10 (98%), CD22 (90%), CD38 (99%)	/	/	CD22<br>S-HCL-1 (BD)	CD19<br>J3.119 (BC)	CD10<br>HI10a (BD)	CD38<br>HB7 (BD)
6	CD45 (12%), CD10 (87%), CD19 (79%), CD22 (86%), CD34 (70%) HLA-DRII (81%)	/	/	CD22<br>S-HCL-1 (BD)	CD19<br>J3.119 (BC)	CD10<br>HI10a (BD)	CD38<br>HB7 (BD)
7	CD2 (89%), cyCD3 (94%), CD5 (93%), CD7 (98%), CD10 (49%), CD33 (81%), CD34 (89%), CD45RA (99%), CD123 (56%)	CD7<br>M-T701 (BD)	/	CD5<br>BL1a (BC)	cyCD3<br>UCHT1 (BC)	CD34<br>581 (BD)	sCD3<br>SK7 (BD)
8	CD2 (76%), CD7 (94%), CD13 (98%), CD10 (80%), cyCD3 (83%), cyCD79 (80%)	CD7<br>M-T701 (BD)	/	CD13<br>L138 (BD)	cyCD3<br>UCHT1 (BC)	CD10<br>HI10a (BD)	sCD3<br>SK7 (BD)
9	CD13 (100%), cyCD13 (100%), CD15 (55%), CD33 (100%), CD34 (100%), CD38 (100%),CD117 (100%), cMPO (100%)	/	/	CD361<br>MEM-216<br>(Exbio)	CD34<br>581 (BC)	CD117<br>104D2 (BD)	CD43<br>1G10 (BD)
10	CD34 (96%), CD33 (98%), CD13 (54,5%), CD117 (47%), CD38 (40,5%)	/	/	CD33<br>P67.6 (BD)	CD11b<br>Bear1 (BC)	CD117<br>104D2 (BD)	CD43<br>1G10 (BD)
11	CD7 (80%), CD11b (83%), CD13 (96%), cCD13 (66%), CD19 (51%), CD33 (96%), CD34 (100%), CD38 (100%), CD71 (93%), CD117 (100%), cyMPO (100%), HLA DR (92%)	/	/	CD33<br>P67.6 (BD)	CD11b<br>Bear1 (BC)	CD15<br>HI98 (BD)	CD43<br>1G10 (BD)
12	CD13 (90%), CD33 (99%), CD117 (99%), CD65 (86%), CD7 (99%), HLA DR2 (97%), CD34 (98%), CD38 (98%)	/	/	CD33<br>P67.6 (BD)	CD34<br>581 (BC)	CD117<br>104D2 (BD)	CD43<br>1G10 (BD)
13	CD13 (100%), CD33 (100%), CD65 (100%), CD117 (100%) et CD11c (100%)	/	/	CD33<br>P67.6 (BD)	CD11b<br>Bear1 (BC)	CD117<br>104D2 (BD)	CD43<br>1G10 (BD)
14	CD13 (100%), CD33 (100%), CD117 (100%), CD34 (100%), CD38 (100%), CD123 (89%), cyMPO (100%), cyCD13 (100%)	/	/	CD33<br>P67.6 (BD)	CD34<br>581 (BC)	CD117<br>104D2 (BD)	CD43<br>1G10 (BD)
15	CD34 (100%), CD33 (50%), CD38 (100%), HLA DR (100%), CD99 (50%)	/	CD43<br>1G10 (BD)	CD33<br>P67.6 (BD)	CD34<br>581 (BC)	HLA-DR<br>REA805 (MB)	/

BC, Beckman Coulter; BD, Becton Dickinson; MB, Miltenyi Biotec.
